# Beyond Immune Cell Migration: The Emerging Role of the Sphingosine-1-phosphate Receptor S1PR4 as a Modulator of Innate Immune Cell Activation

**DOI:** 10.1155/2017/6059203

**Published:** 2017-08-07

**Authors:** Catherine Olesch, Christian Ringel, Bernhard Brüne, Andreas Weigert

**Affiliations:** Institute of Biochemistry I, Faculty of Medicine, Goethe University Frankfurt, 60590 Frankfurt, Germany

## Abstract

The sphingolipid sphingosine-1-phosphate (S1P) emerges as an important regulator of immunity, mainly by signaling through a family of five specific G protein-coupled receptors (S1PR1–5). While S1P signaling generally has the potential to affect not only trafficking but also differentiation, activation, and survival of a diverse range of immune cells, the specific outcome depends on the S1P receptor repertoire expressed on a given cell. Among the S1PRs, S1PR4 is specifically abundant in immune cells, suggesting a major role of the S1P/S1PR4 axis in immunity. Recent studies indeed highlight its role in activation of immune cells, differentiation, and, potentially, trafficking. In this review, we summarize the emerging data that support a major role of S1PR4 in modulating immunity in humans and mice and discuss therapeutic implications.

## 1. Introduction

Lipids do not only serve as energy storage and constitute a major part of cellular membranes but also are important signaling molecules that have potent immunoregulatory function. Some well-known examples of immunoregulatory lipids are unsaturated fatty acid derivatives such as prostanoids, leukotrienes, and other eicosanoids, which play crucial roles in acute and chronic inflammatory disease settings [1]. Also, the lipid class of sphingolipids harbors signaling molecules with potent immunomodulatory properties, the most prominent among them being sphingosine-1-phosphate (S1P) [2]. Research on the role of bioactive lipids such as S1P has particularly in the last decades begun to gather steam, when these lipids were tied to specific GPCRs, in the case of S1P to a family of five GPCRs (S1PR1–5) [3, 4]. Since then, S1P has been shown to play important roles in regulating cell biology and organismal homeostasis by promoting cell survival, migration, and differentiation. Moreover, it emerged as an important player in immunity and inflammation. S1P not only promotes the egress of lymphocytes from secondary lymphoid organs into the bloodstream [5] but also modulates the cytokine profile of innate and adaptive immune cells, thereby affecting physiological and pathological inflammation [2]. A multitude of the immunomodulatory effects of S1P have been attributed to signaling through S1PR1, whereas the contribution of other S1P receptors remains largely obscure. S1PR4 is particularly expressed by immune cells and may therefore be critically involved in immunomodulation by S1P. In this review, we therefore summarize the current knowledge about S1PR4 and discuss therapeutic implications of interfering with its signaling, particularly in chronic inflammatory disease settings.

## 2. S1P and Its Receptors in Immunity

The sphingolipid S1P is a bioactive signaling molecule that plays a major role in physiological as well as pathophysiological settings, regulating survival, proliferation, migration, and cell type-specific functional responses. In the immune system, S1P affects mainly lymphocyte trafficking, but it is also involved in immune cell development and modulates their adaption to activating stimuli. S1P is produced via metabolic breakdown of the ubiquitous membrane lipid sphingomyelin first to ceramide, which is further deacetylated to sphingosine. Sphingosine can finally be phosphorylated to S1P by two sphingosine kinases (SPHK1 and SPHK2), with different subcellular localization and divergent functional roles [6]. Upon formation, S1P acts as an intracellular or extracellular signaling molecule until it is dephosphorylated by S1P phosphohydrolases 1 and 2 or degraded by S1P lyase (SPL) [7].

The importance of S1P for organismal development and homeostasis is underlined by findings that mice deficient in both SPHK 1 and 2 die prenatally from hemorrhage, indicating a dysfunctional development of the vascular system [8]. A few intracellular targets of S1P signaling that are relevant for inflammatory events have been identified, including TNF-*α* receptor-associated factor 2 (TRAF2), an E3 ubiquitin ligase of the nuclear factor “kappa-light-chain-enhancer” of activated B-cells (NF-*κ*B) pathway [9], inhibitor of apoptosis 2 (cIAP2), which promotes polyubiquitination of interferon regulatory factor-1 to enhance chemokine expression [10], class I histone deacetylases HDAC1 and HDAC2 [11], and the mitophagy receptor prohibitin 2 [12]. Here, S1P primarily acts as a cofactor. Besides, ceramide synthase 2 is directly inhibited by S1P [13], which may either enhance or suppress inflammation [14, 15]. Despite these intracellular targets, S1P appears to exert the majority of its functions in immunity by activating its 5 specific G protein-coupled receptors. To enable this, S1P, once being generated intracellularly, can be exported to the extracellular space by a number of transporters including members of the ABC transporter family [16–18] as well as spinster homolog 2 (SPNS2) [19]. Among these, SPNS2 appears to be essential for S1P-dependent immune regulation. SPNS2-deficient mice showed reduced circulating S1P levels and lymphopenia, which translated into reduced severity of airway inflammation, delayed-type contact hypersensibility, dextrane sulphate sodium-induced colitis, experimental autoimmune encephalopathy (EAE), and collagen-induced arthritis, likely due to suppressed lymphocyte trafficking [20]. Once released into the circulation, S1P is bound by high-density lipoprotein (HDL)-associated apolipoprotein M (ApoM) or albumin. Different S1P chaperones appear to confer divergent biological functions to S1P, since ApoM-bound S1P does not affect lymphocyte trafficking but restrains lymphopoiesis in the bone marrow. Limiting S1P-bound ApoM levels increased EAE severity [21]. Irrespective of the mode of transport, S1P may couple at least to S1PR1 in an autocrine fashion that does not involve transport into the extracellular space. A recent report suggests that S1P accesses S1PR1 laterally by inserting into the plasma membrane [22]. It remains unknown if this mode of activation applies also for other S1PRs. Thus, S1P signaling is determined by its localization and mode of presentation. Another layer of complexity is added by cell type-specific expression of different S1PR profiles, since individual S1PRs couple to different heterotrimeric G proteins and therefore are able to elicit diverse and sometimes antithetic responses. While S1PR1, 2 and 3 are expressed ubiquitously, S1PR4 and 5 show tissue-specific distribution. S1PR4 is exclusively found in hematopoietic tissues under basal conditions [23], whereas S1PR5 expression is restricted to natural killer (NK) cells [24], dendritic cells (DCs) [25], the central nervous system [26], endothelial cells [27], and certain cancer cells [28, 29], indicating specialized functions of these two S1PRs.

In homeostasis, S1P levels in tissues are kept below concentrations that are required to activate S1PR signaling [7]. High concentrations of S1P in a nanomolar to micromolar range are solely detected in blood and lymph, the majority being bound to albumin or HDL [7, 21]. This centralized distribution of S1P is critical for its main biological functions, the maintenance of vascular integrity, and white blood cell trafficking [30]. However, under inflammatory conditions, extravascular S1P levels may rise due to cellular signaling activating SPHK1-dependent S1P secretion, or inflammation-induced cell death and subsequent release of active SPHK2 to the extracellular space [31]. By virtue of these mechanisms, S1P plays a pivotal role in inflammation that reaches beyond its homeostatic function of immune cell trafficking. The decisive role of S1P in immune cell trafficking was discovered when the immunosuppressive agent FTY720 was found to induce S1PR (S1PR1, 3–5) internalization in T cells to render them unresponsive to the S1P gradient towards the circulation, thereby trapping them in thymus and secondary lymphatic organs [32, 33]. Generally, S1PR1 is strongly upregulated during T cell development to enable the egress of mature T cells from thymus into blood [5]. Once in the circulation, S1PR1 is internalized due to high blood S1P concentrations, thereby making T cells responsive to other chemotactic signals and enabling them to extravasate into peripheral tissues for surveillance. There, S1PR1 relocates to the plasma membrane due to the absence of its ligand, and T cells are ready to traffic back into the circulation following the S1P gradient. The establishment of tissue-resident memory T cells therefore requires permanent downregulation of S1PR1 [34].

This pattern of S1P-dependent migration has been described for most immune cell subsets, although the receptors used can differ. B cells utilize S1PR1 and 3 to localize to their proper locations in secondary lymphoid organs [5, 35], and NK cell migration towards S1P depends on S1PR5 [24]. Circulating myeloid cells such as monocytes respond to S1P mainly through S1PR3 [36] or S1PR5 [37]. The situation is slightly more complex for myeloid cells such as DCs and macrophages, which usually reside in tissues and are only supposed to emigrate into the circulation upon activation and antigen capture. In these cells, responsiveness to S1P is regulated by the S1PR repertoire expressed prior and subsequent to activation. Immature DCs express predominantly S1PR2 and 4, with the notion that S1PR2 usually counteracts the promigratory function of S1PR1 by signaling towards chemorepulsion. During maturation after antigen uptake, S1PR1 and 3 are profoundly induced, now enabling mature DCs to emigrate into the lymphatic system to present the captured antigens to lymphocytes [38]. A similar scenario applies to macrophages in different phases of inflammation. While S1PR2 dominates in proinflammatory cells, S1PR1 is upregulated during resolution of inflammation to enable the emigration of macrophages from the site of inflammation [39, 40]. Interestingly, this manner of myeloid cell trafficking is also hijacked by pathogens as migration of infected monocytes and DCs to draining lymph nodes in an S1PR1-dependent manner was connected to spreading infection with *Y. pestis* [41].

Besides regulating immune cell migration, S1P influences immune cell survival, differentiation, and activation. These topics have been covered by recent reviews to which we would like to refer [6, 30, 40, 42]. To name a few significant findings, S1PR1 signaling on myeloid cells increases tumor-promoting inflammatory cytokine production [43] and shapes T cell activation by promoting T helper 17 (Th17) and limiting regulatory T cell (Treg) polarization [44, 45]. Compared to the well-characterized role of S1PR1 in these processes, the function of S1PR4 is so far underappreciated. Recent reports suggest its potential involvement in chronic inflammatory responses, which is discussed in the following paragraphs.

## 3. S1PR4 Signaling

Signaling pathways induced downstream of S1PR4 upon ligation by S1P are largely unexplored, although early studies at least pointed towards the specific G proteins that are activated in response to triggering this seven-transmembrane domain receptor. S1P was first shown to couple to S1PR4 (then known as EDG-6) in 2000 [4], confirming previous predictions of EDG-6 as a putative S1P receptor [23]. In early signaling studies, S1PR4-transfected HEK293 cells showed extracellular-signal regulated kinase 1/2 (ERK1/2) activation upon S1P stimulation, which was pertussis toxin-sensitive, indicating that S1PR4 coupled to G*α*_i_ [4]. Subsequently, S1PR4 was overexpressed in CHO cells to further investigate G protein coupling [46]. Hereby, S1PR4 was shown to signal via G*α*_i_ and G*α*_12/13_ but not G*α*_q_ and G*α*_15/16_ [46], although the tandem genomic arrangement of S1PR4 and G*α*_15/16_ and frequent cellular coexpression were suggestive of a functional interaction [47, 48]. Furthermore, coupling to G*α*_s_ was excluded by the inability of S1PR4-overexpressing CHO cells to increase cAMP production upon S1P stimulation [46]. Signaling through S1PR4 dependent on pertussis toxin and therefore G*α*_i_ induced phospholipase C activity and a subsequent increase in cytosolic Ca^2+^, which was attributed to the *βγ* subunits of this heterotrimeric G protein [46, 49]. Interestingly, S1P coupling to S1PR4 activated the small G protein RhoA, likely in a G*α*_12/13_-dependent manner, and induced cytoskeletal rearrangements and cell rounding. RhoA activates cofilin via RhoA kinase (ROCK) and LIM domain kinase, which is involved in actin nucleation and severing of actin fibers, as well as myosin light chain, which promotes actin contractility, both of which may be involved in cell rounding downstream of S1PR4 [50]. These data suggested a major influence of S1PR4 signaling on components that mediate cytoskeletal (re-)arrangement.

Another set of data that did not employ S1PR4 overexpression strategies confirmed the impact of S1PR4 signaling on activation of these pathways, specifically on ROCK activation [51]. TGF-*β* induced S1PR4 upregulation was observed in myoblasts. S1PR4 is usually not expressed outside the immune system but may be induced in nonhematopoietic cells under certain conditions. Signaling through S1PR4 in myoblasts involved activation of ROCK2, leading to phosphatase and tensin homolog (PTEN) phosphorylation and subsequent inhibition of protein kinase B (PKB/AKT) signaling. Consequentially, inhibition of AKT by S1PR4 induced cell death in myoblasts, which is a known detrimental function of TGF-*β* in wound healing [51]. Interestingly, S1PR4 in these cells did not affect ERK1/2 activation, which might have counteracted the negative effect of S1PR4 on myoblast survival, as indicated for breast cancer cells [52]. In these cells, S1PR4 signaling stimulated ERK1/2 phosphorylation by a pathway involving tyrosine phosphorylation of human epidermal growth factor receptor 2 (HER2) [52].

Also, in immune cells, S1PR4 signaling was connected to pathways altering actin dynamics. In human plasmacytoid dendritic cells (pDCs), triggering S1PR4 signaling with S1P or a specific S1PR4 agonist prevented the activation-induced internalization of the inhibitory cell surface receptor leukocyte immunoglobulin-like transcript (ILT7). ILT7 internalization in this context was restored with antagonists of both RhoA and ROCK, indicating that a G*α*_12/13_, RhoA, and ROCK-dependent signaling pathway activated by S1PR4 ligation prevented ILT7 internalization [53]. Since receptor endocytosis is highly dependent on actin dynamics [54], a role of altered actin dynamics downstream of S1PR4 in modulating ILT7 internalization appears rational. S1PR4 signaling furthermore facilitated AKT activation to promote cytokine production in human macrophages [55]. In this setting, again, receptor trafficking downstream of S1PR4 was affected. S1P derived from dying tumor cells promoted the shuttling of the nerve growth factor (NGF) receptor tropomyosin receptor kinase A (TRKA) from intracellular vesicles to the plasma membrane, where constitutively produced NGF activated AKT [55]. TRKA shuttling by S1PR4 required the activation of a proto-oncogene tyrosine-protein kinase Src family protein. Src family proteins are associated with the regulation of actin dynamics via phosphorylation of Rho guanine nucleotide exchange factors (GEFs) that consequently activate Rho and ROCK [56]. How S1PR4 activates Src remains elusive. However, Src family activity by GPCRs can be triggered by G*α*_i_, independently of G proteins by direct association with the GPCR or via transactivation of receptor tyrosine kinases [57, 58].

**Figure 1:**
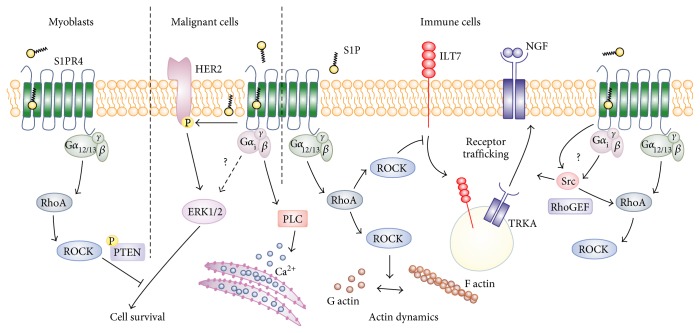
S1PR4 signaling. S1PR4 couples to G*α*_i_ and G*α*_12/13_ to regulate cell survival, actin dynamics, and receptor trafficking. G*α*_i_ activation downstream of S1PR4 mediates cytosolic Ca^2+^ increases via PLC and directly or indirectly induces ERK1/2 and Src signaling. G*α*_12/13_ predominantly triggers RhoA and subsequent ROCK activity. Cell type-specific signaling events are indicated. Details can be found in the main text. ERK: extracellular-signal regulated kinase; HER2: human epidermal growth factor receptor 2; ILT7: immunoglobulin-like transcript 7; NGF: nerve growth factor; PLC: phospholipase C; PTEN: phosphatase and tensin homolog; RhoGEF: Rho guanine nucleotide exchange factor; ROCK: RhoA kinase; TRKA: tropomyosin receptor kinase A.

In conclusion, S1PR4 couples to G*α*_i_ and G*α*_12/13_ to induce MAPK activity but predominantly activates RhoA/ROCK to affect actin dynamics in different cell types (Figure 1). This process then regulates trafficking of other receptors, which appears to be a common feature underlying the biological functions of S1PR4 at least in immune cells. In contrast, the impact of S1PR4 signaling on cell survival is likely cell type-specific. In the following paragraphs, we describe the immunological consequences of S1PR4 signaling, for most of which exact signaling pathways downstream of S1PR4 so far remain elusive.

## 4. S1PR4 and Immune Cell Trafficking

The role of S1PR4 in immune cell trafficking is at present controversially discussed. While its impact is nowhere as prominent as that of S1PR1, although expression levels in lymphocytes appear to be similar, neutrophil homeostasis is prominently regulated by S1PR4, which may involve modulation of neutrophil migration. As T cells have long been in the focus of research on the role of S1P in cell motility, with several studies showing how S1P controls the ability of T cells to leave lymph nodes via S1PR1 [5, 59], early studies on the influence of S1PR4 on migration also focused on T cells. The already high expression of S1PR4, compared to S1PR1, in these results was supplemented by additional overexpression of S1PR4 on Jurkat T cells, and primary mouse splenocytes, which increased their spontaneous motility. This was, however, not enhanced by S1P stimulation, indicating either sufficient endogenous S1P production by these cells or that enhanced motility was an artifact of overexpression and S1PR4 signaling per se was not involved [46]. These notions were supported later by a following study showing that neither transgenic expression of S1PR4 in murine T cell lines nor inhibition of S1PRs with FTY720 in primary CD4^+^ splenocytes changed chemotaxis towards S1P [60]. On a contrary note, Matsuyuki et al. reported in 2006 that the same murine T cell lines showed chemotaxis towards S1P, which was inhibited by the nonselective S1PR superagonist FTY720 [61]. They argued that this effect was at least in part attributable to S1PR4 based on its prominent expression among S1PRs in these cells. In addition, they also demonstrated association of S1PR4 with the other highly expressed S1P receptor, S1PR1, proposing a functional codependency of the two receptors. Such a codependency of S1PR4 and S1PR1 was also suggested by a study utilizing human B cell lines. However, the authors reported that endogenous S1PR4 had no impact on B cell line migration, but overexpression of S1PR4 mildly reduced S1PR1-dependent migration of B cell lines [62]. In conclusion, studying immune cell migration using cell lines overexpressing S1PR4 did not provide sufficient data to clearly delineate the role of this receptor in immune cell migration.

More conclusive data emerged from studies using S1PR4-deficient animals. Using such animals, Schulze et al. showed that motility of murine CD4^+^ and CD8^+^ T cells was mildly enhanced *in vitro*, which was confirmed for CD8^+^ T cells *in vivo*, supporting the abovementioned findings of a negatively modulating role of S1PR4 in lymphocyte migration [63]. Besides, this study revealed a rather prominent function for S1PR4 in DC trafficking. Loss of S1PR4 in a model of allergic airway disease caused a marked enrichment of DCs in lymph nodes [63]. There are a number of possible explanations for this phenomenon. First, DC trafficking towards lymph nodes is regulated via chemokine signaling of CCL19 and CCL21 through CCR7 that is upregulated on DCs following their activation. Activation of DCs likewise increases S1PR1 expression and decreases S1PR2, thereby enhancing their migratory capacity [64, 65]. Therefore, it is conceivable that S1PR4 antagonizes S1PR1, and that its depletion thereby increases DC numbers in draining lymph nodes. Alternatively, egress of DCs from lymph nodes could be S1PR4 dependent or S1PR4 signaling might limit the DC life-span similar to the situation described above for myoblasts [51]. A promigratory role of S1PR4 on DCs might be supported by the observation that common dendritic precursors (CDPs) showed a strong chemotactic response towards S1P that was strictly dependent on S1PR4, since this response was absent in cells derived from S1PR4 knockout animals.

Also, neutrophils are discussed to migrate towards S1P via S1PR4. Initial studies showed that S1P inhibits neutrophil migration towards IL-8 and fMLP [66, 67]. This inhibition did not occur after addition of S1P precursors or analogs and therefore was most likely regulated by one of the S1P receptors, of which neutrophils express S1PR1, S1PR4, and S1PR5. However, the experimental design of these studies did not allow excluding indirect effects of S1P on other cells such as endothelial cells. Despite these inhibitory effects on neutrophil migration, S1P treatment alone was sufficient to significantly increase neutrophil migration, especially for neutrophils originating from patients suffering from pneumonia [67]. Moreover, in mice immunized with ovalbumin, the S1PR superagonist FTY720 did not inhibit migration of neutrophils towards the site of inflammation, but abrogated their migration from inflamed tissues towards draining lymph nodes. In this study, S1PR4 was also identified as the only S1PR neutrophils upregulate upon stimulation [68]. Together, these findings suggest S1P as a regulator of neutrophil motility, putatively through S1PR4 and especially during inflammation.

In a genome-wide association study of low-frequency coding variants with hematologic parameters, a S1PR4 variant was identified as a marker for neutrophil count [69]. The observed S1PR4 variant was correlated to a significant reduction in the number of circulating neutrophils. In confirmation of this observation in large human cohorts, a significant reduction of neutrophil count was observed when analyzing large cohorts of S1PR4 knockout mice and zebrafish [69]. A reduced recruitment from the primary site of granulopoiesis was ruled out by the observation that neutrophils did not accumulate in the bone marrow of S1PR4 knockout mice. The authors also observed a reduction of CD62L on neutrophils, which is shed upon their activation and mediates their interaction with the endothelium, which might suggest enhanced migration of neutrophils into peripheral tissues when S1PR4 is lacking. However, reduced numbers of tissue neutrophils in liver and lungs of S1PR4-deficient mice that were comparable to the changes in blood levels were noted, suggesting that this loss of CD62L was not due to activation upon invasion [69]. Allende et al. showed a similar reduction of CD62L on neutrophils in a mouse strain deficient in SPL and went on to show that this reduction was due to diminished expression [70]. They observed that, in contrast to S1PR4-deficient animals, SPL-deficient animals showed a strong increase of circulating neutrophils and proinflammatory cytokines such as CCL2 and IL-6. They also observed a marked reduction of chemotaxis towards fMLP, which is in line with previous findings described above. This phenotype, with exception of the reduced CD62L expression, was rescued when S1PR4 was depleted together with SPL in double transgenic animals [70]. This observation likely excludes reduced CD62L expression as the mechanistic explanation for reduced neutrophil counts in S1PR4-deficient animals. However, it strengthens the finding that S1PR4 affects neutrophil homeostasis critically, both under steady-state and hyperinflammatory conditions. Mechanistically, Allende et al. argued that under conditions of single depletion of SPL, the inability of CD62^low^ neutrophils to migrate into tissues deprived tissue-resident macrophages and DCs of suppressive signals resulting from dying neutrophils, causing these macrophages or DCs to produce IL-23 and activate the IL-23/IL-17 axis, which induces granulopoiesis [71]. This was corroborated by an observed increase in Th17 cells and could explain the increased granulopoiesis as well as the high levels of proinflammatory cytokines in SPL-deficient mice [70]. Based on this hypothesis, an additional knockout of S1PR4 might not affect the neutrophils directly, but rather other cells of the immune system that produce Th17 promoting cytokines. The most elegant explanation would tie S1PR4 to the IL-23/IL-17 axis, which was indeed observed when S1PR4-deficient mice were challenged with several diseases [63]. This explanation seems plausible, although the mechanistic detail including CD62L appears to be irrelevant, since both single SPL and S1PR4 depletion were associated with CD62L, although neutrophil counts were affected contrarily. At least two explanations therefore remain. S1PR4 may potentially promote the life-span of mature neutrophils without affecting their generation from precursors in the bone marrow, which would result in reduced peripheral neutrophil counts when S1PR4 is lacking. Another more likely explanation is that S1PR4 directly promotes Th17 polarization and therefore granulopoiesis as part of a complex signaling network.

In conclusion, S1PR4 appears to play a minor regulatory role in immune cell migration, with the exception of DCs and DC precursors. Apart from these relatively clear indications, contradictory or controversial findings for a role in lymphocyte or neutrophil migration are likely a result of complex cellular systems or the use of unspecific receptor agonists/antagonists. Future research on the role of S1PR4 in immune cell motility therefore requires the use of the recently developed S1PR4-specific agonists and antagonists [72–75].

## 5. S1PR4 and Immune Cell Differentiation

S1PR4-deficient mice and zebrafish are born at normal frequencies and do not show an obvious abnormal phenotype when they remain unchallenged [76, 77]. This indicates a negligible influence of S1PR4 during embryonic development and on the development of individual immune cell populations, although S1PR4 is highly expressed by hematopoietic stem cells [78, 79]. However, studies which more closely investigated the immune cell composition in S1PR4-deficient mice, reported an impact of S1PR4 on megakaryocyte and DC differentiation under basal conditions [76].

When looking at megakaryocyte differentiation from human progenitors, S1PR4 expression was increased along the line of progenitor differentiation into mature megakaryocytes and was expressed in mature megakaryocytes of mice as well [76]. Furthermore, S1PR4-deficient mouse bone marrow contained a substantial number of aberrant megakaryocytes. While reduced formation of proplatelets from S1PR4-deficient mouse bone marrow was observed *in vitro*, the number of platelets under basal conditions was unchanged. Nevertheless, platelet recovery after antibody-induced platelet deletion was delayed when S1PR4 was lacking. Moreover, overexpression of S1PR4 alone was sufficient to induce differentiation of human erythroleukemia (HEL) cells to megakaryocytes. This was accompanied by the upregulation of megakaryocyte and platelet markers such as CD41 and the platelet-specific ADP receptor P2Y12, indicating that S1PR4 signaling in this context alters genetic programs to modulate cell identity. Mechanistic explanations connecting S1PR4 to such processes are so far elusive. However, apart from altering gene expression, platelet formation also requires alterations of the cytoskeleton [80], which, as outlined above, is a target of S1PR4 signaling. The specific pathways downstream of S1PR4 that promote proplatelet formation may be the object of future investigations.

Within the DC lineage, murine pDCs nearly exclusively express S1PR4 among S1P receptors [81]. In a study initially designed to look at S1P-dependent migration of murine pDCs, it was noted that S1PR4-deficient mice specifically lack the subpopulation of migratory CD4^−^ pDCs in blood and primary as well as secondary hematopoietic organs [82]. Frequencies of other immune cells were not affected. While S1PR4 did not modify pDC migration, it appeared to specifically promote the migration of common dendritic cell precursors (CDPs) towards S1P *in vitro*. This was correlated with an accumulation of CDPs in murine S1PR4-deficient bone marrow compared to WT bone marrow [82]. A possible explanation for this correlation is the observation that pDCs develop in regions with high oxygen in the bone marrow, indicated by the fact that hypoxia-inducible factor-1 limits pDC development [83], which are likely enriched in vasculature and therefore in contact with the high levels of S1P in the circulation. CDPs might follow the S1P gradient into these regions via S1PR4 to differentiate to pDCs. Regardless of this hypothesis, differentiation of pDC precursors to pDCs was also S1PR4-dependent in fms-like tyrosine kinase 3-ligand- (Flt3-L-) driven *in vitro* assays where S1P and oxygen gradients are unlikely. As discussed above, S1PR4 couples to G*α*_i_ to activate growth-promoting signals such as ERK [4] and PKB/AKT that are also activated downstream of Flt3. Since Flt3 signaling on CDP is critical for pDC development, a positive modulation of the Flt3 signaling pathway by S1PR4 appears as another possible explanation for the reduction of pDCs in S1PR4-deficient mice.

In conclusion, S1PR4 modulates the differentiation of a selected set of immune cells, likely by modulating primary signals involved in their generation or by regulating the migration of progenitors. Based on these studies, interfering with S1PR4 pharmacologically might affect platelet dynamics and limit pDC generation, both of which are relevant processes in human disease [76, 84].

## 6. S1PR4 and (Myeloid) Immune Cell Activation

S1PR4 was initially described to directly regulate cytokine production by T cell lines. However, in these studies, S1PR4 was overexpressed [60]. Subsequent studies and our own unpublished observations indicate that S1PR4 expression of lymphocytes does not affect their activity directly, although lymphocytes show high levels of S1PR4 expression [63, 85]. Instead, several publications point towards an indirect regulation of lymphocyte function by S1PR4 [53, 63, 85]. Thereby, S1PR4 signaling seems to mainly effect cytokine production of antigen-presenting cells (APCs) including DCs and macrophages to shape T cell effector function. Cytokines released by APCs are the polarizing signal during T cell activation, which is one of the three signals provided by phagocytes to facilitate proper T cell priming [86]. The other two are the presentation of antigens via major histocompatibility complex (MHC) or related molecules and cell surface expression of costimulatory molecules, which occur as a consequence of APC activation/maturation. APC activation/maturation was found to be S1PR4-independent in several model systems. Expression of activation markers such as MHCII and costimulatory molecules by murine LPS-stimulated BMDCs, human monocyte-derived DCs activated by apoptotic cells, and CpG-activated pDCs was unchanged when S1PR4 was depleted or antagonized [53, 63, 85].

A number of recent publications demonstrated that shaping Treg and, most prominently, Th17 cell polarization by cytokines released from DCs and macrophages is strongly dependent on S1PR4 signaling. Whereas the release of IL-10 and TGF-*β* shapes the phenotype of Tregs, which are known to inhibit, among others, cytotoxic T cell function and proliferation, IL-6, TGF-*β*, IL-23, and IL-1*β* are required for Th17 differentiation [87]. Murine DCs required S1PR4 for efficient IL-6 secretion and subsequent Th17 differentiation. When S1PR4-deficient bone marrow-derived DCs (BMDCs) were stimulated with LPS, they secreted less IL-6 and coculture of these BMDCs with antigen-specific T cells decreased T cell-specific IL-17 production [63]. IL-6 levels were also reduced in serum of S1PR4-deficient mice suffering from inflammatory DSS-induced colitis, which is Th17-dependent [88]. These mice lost less weight and therefore had a less severe disease progression [63]. Consequently, S1PR4 was proposed to enhance Th17-driven inflammation by polarizing Th cells towards the Th17 lineage, which was dependent on DCs releasing the Th17 polarizing cytokine IL-6. A role of S1PR4 in IL-6 production was also confirmed for macrophages. Activation of S1PR4 signaling on human and murine tumor-associated macrophages (TAMs) by apoptotic tumor cell-derived S1P leads to the production of tumor-promoting cytokines, including IL-6 and IL-10. This depended on the shuttling of TRKA to the cell surface [55]. In conclusion, S1PR4 appears to be critically involved in Th17 polarization, probably via affecting IL-6 release. However, mechanistic details connecting S1PR4 signaling to IL-6 release are sparse. Nevertheless, these data suggest that S1PR4 harbors the potential to be a feasible target for affecting disease progression of Th17-driven immune-mediated diseases such as psoriasis, asthma, and inflammatory bowel disease [89].

Besides promoting Th17 polarization, S1PR4 also indirectly affected other T cell subsets by regulating cytokine release from APCs. *In vitro* studies demonstrated that S1P produced by apoptotic tumor cells triggered S1PR4-dependent production of IL-27 by human DCs. IL-27 upregulated CD69 on a subset of Tregs (CD39^+^) that were subsequently able to efficiently suppress cytotoxic lymphocytes most likely by the release of adenosine [85]. Other functions of S1PR4 likely also affect cytotoxic lymphocyte activity directly or indirectly. Activated pDCs released lower levels of IFN-*α* when S1PR4 was stimulated simultaneously [53]. IFN-*α* production during the immune response against viral infections is the primary function of pDCs, which is required to activate cytotoxic lymphocytes. It was shown that tumor-associated pDCs are impaired in IFN-*α* production leading to Treg expansion and breast cancer progression [90]. Accordingly, S1PR4-stimulated pDCs promoted the expansion of IL-10-producing T cells, likely Tregs [53]. Elucidating the role of S1PR4 during tumor development therefore appears promising.

Together, the studies summarized in this paragraph strongly suggest an immunomodulatory role for the immune cell-specific S1PR4 during inflammation and a potential protumoral role during cancer progression, both by modulating T cell function through myeloid cell activation. However, further studies using S1PR4-deficient mice and/or specific S1PR4 agonists/antagonists are needed to shed light onto S1PR4-mediated myeloid cell/T cell interactions.

## 7. Conclusions

**Figure 2:**
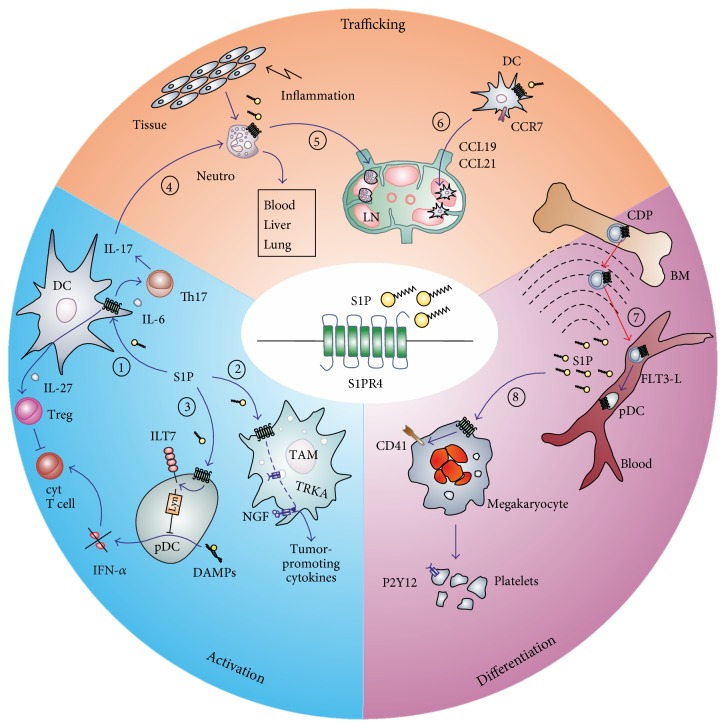
Impact of S1PR4 on immune cell activation, trafficking, and differentiation. (1) S1P triggers the S1PR4-dependent production of IL-27 by human DCs, which enables Tregs to efficiently suppress cytotoxic CD8^+^ T cells. (2) S1PR4 activation induces translocation of TRKA to the cell surface to enable the production of tumor-promoting cytokines (IL-6, IL-10) by macrophages. (3) S1PR4 activation preserves surface expression of the human pDC-specific inhibitory receptor ILT7 leading to decreased DAMP-induced IFN-*α* secretion and reduced cytotoxic T cell activation by human pDCs. (4) Enhanced S1PR4-dependent IL-17 production increases neutrophil numbers in blood, liver, and lung of mice putatively by enhancing granulopoiesis. (5) Activation of S1PR4 on neutrophils enhances neutrophil trafficking from the inflamed tissue to the draining lymph node. (6) Activation of S1PR4 on DCs leads to an enrichment of DCs in lymph nodes among others regulated by CCL19 and CCL21 through CCR7. (7) S1PR4 on CDPs within the bone marrow stimulates their trafficking towards well-perfused areas following the S1P gradient, where they differentiate to pDCs under the influence of FLT3-L. (8) S1PR4 signaling during megakaryocyte development promotes their differentiation and the formation of platelets accompanied by the upregulation of megakaryocyte and platelet markers such as CD41 and the platelet-specific receptor P2Y12. DAMP: danger-associated molecular pattern; DC: dendritic cell; FLT3-L: fms-like tyrosine kinase 3-ligand; ILT7: inhibitory receptor Ig-like transcript 7; pDC: plasmacytoid DC; TRKA: tropomyosin receptor kinase A.

The role of S1PR4 in inflammation is slowly being unraveled. While signaling through S1PR4 appears to rather mildly impact immune cell migration, as opposed to the other members of this family, and influences differentiation of a very limited number of immune cell subsets, regulation of cytokine production by myeloid cells appears to be most influential (Figure 2). Mechanistic details connecting S1PR4 signaling to these features are sparse but may involve transactivation of other receptors on immune cells by regulating their subcellular localization. A pathological role of S1PR4 appears most obvious under conditions of chronic inflammation. Its potency to reduce neutrophil numbers in mice and humans and to limit Th17 polarization may render it an attractive target to interfere with diseases characterized by these features [91, 92].

Among diseases with a clear Th17 etiology are psoriasis and rheumatoid arthritis [93]. Treatment with S1PR4 antagonists, thereby reducing IL-17 production, may therefore be of interest in these entities. Although S1PR4 deficiency showed a major improvement of DSS-induced colitis, inflammatory bowel disease (IBD) in humans does not appear to be strictly Th17-dependent [93]. In fact, neutralizing IL-17 has even shown an adverse effect in patients with Crohn's disease [94], probably limiting the applicability of targeting S1PR4 to treat IBD. Another counterargument could be made by the availability of IL-17 targeting biologicals, raising the question what benefit targeting S1PR4 might have beyond targeting IL-17 directly [95]. Besides the limited costs of a small molecule S1PR4 inhibitor, it is important to stress that targeting a single inflammatory mediator in chronic inflammatory diseases may lead to therapeutic resistance, which is well known for TNF-*α* neutralizing biologicals [96]. Inhibiting S1PR4 may target more than one inflammatory mediator at once, for example, IL-6 and IL-17 [55, 63]. To understand the full potential of S1PR4 modulation, systems biology approaches need to be undertaken to unravel cytokine and, importantly, chemokine networks under the control of S1PR4. In contrast to contributing to inflammatory mediator production, S1PR4 was also involved in producing the anti-inflammatory cytokine IL-10 [55]. Moreover, S1PR4 signaling suppressed IFN-*α* production from pDCs, which by itself is a potent driver of certain autoimmune diseases [97]. An involvement of these two mediators in the regulation of chronic inflammation in different disease entities would certainly limit the use of S1PR4 antagonists. On the other hand, the potential to block type I IFN might be beneficial for patients suffering from autoimmune conditions that involve pathogenic type I IFN production, such as systemic lupus erythematosus (SLE) [98]. To envision a use of S1PR4 activators in SLE, future studies need to test if pharmacological overactivation of S1PR4 in disease settings increases the production of inflammatory mediators such as IL-6 and IL-17 that may also be involved in SLE pathogenesis [99, 100]. It is important to stress that pathologically defined autoimmune conditions likely consist of a conglomerate of distinct, etiologically heterogeneous disease subgroups [101]. Therefore, for instance, subgroups of patients with type I IFN-driven SLE may benefit from S1PR4 agonist treatment, whereas other subgroups may not.

In contrast to promoting certain autoimmune conditions, high IFN-*α* production is required for efficient antitumor immunity [102]. Furthermore, IL-17 can promote tumor growth at least in some tumor models [93], and an enhanced neutrophil infiltrate is associated with a poor prognosis among a wide range of human tumors [103]. S1PR4 antagonists might therefore be beneficial in limiting protumor immunity, by reducing the production of IL-17, IL-6, and IL-10 and reducing neutrophil infiltrates, while at the same time promoting antitumor immune responses by increasing type I IFN production. This justifies investigations on the role of this receptor in tumor biology.

Beside these speculations, further investigations are required to clearly dissect the role of S1PR4 in cytokine production in disease models including psoriasis, rheumatoid arthritis, SLE, and cancer. The now available S1PR4-deficient mice and specific S1PR4 agonists and antagonists will be useful in such studies and fuel the identification of specific features of S1PR4 signaling within the multitude of functions mediated by S1P.
